# Mind the Gap: Recurrence of Oligometastatic Breast Invasive Ductal Carcinoma in the Sternal Gap of Two Tangential Photon Fields of Bilateral Whole Breast Irradiation a Decade Later

**DOI:** 10.7759/cureus.46601

**Published:** 2023-10-06

**Authors:** Mary T Mahoney, Noelle Kubinak, Athena Masi, Christopher Lok, Thomas R Eanelli

**Affiliations:** 1 Department of Radiation Oncology, Transitional Year Residency Program, Garnet Health, Middletown, USA; 2 Department of Radiation Oncology, Touro College of Osteopathic Medicine, Middletown, USA; 3 Department of Radiation Oncology, Garnet Health, Middletown, USA

**Keywords:** breast cancer recurrence, cancer recurrence, metastasis directed therapy (mdt), oligometastatic breast cancer, tangential radiation fields, bilateral breast cancer, invasive ductal breast carcinoma

## Abstract

Despite bilateral breast cancer being a rare clinical entity compared to unilateral breast cancer, both share a treatment paradigm of breast-conserving therapy for limited disease and metastasis direct therapy for oligometastatic disease. We present a case of left breast invasive ductal carcinoma in the setting of original bilateral breast cancer, now with oligometastatic recurrence to the soft tissue of the sternum, notably in an area not previously irradiated, over a decade later.

## Introduction

Breast cancer (BC) is still the most common cancer worldwide in women, accounting for approximately 30% of new cancer diagnoses in the United States (US) [1]. Around 85% of BC cases consist of invasive ductal carcinoma (IDC) [1]. Women with primary BC are at increased risk for developing contralateral breast cancer, which can either be concurrent or sequential [2,3]. Unlike unilateral BC (UBC), bilateral BC (BCC) is a much rarer entity with an overall clinical incidence ranging from 1.4% to 11.8% [2,3]. Nevertheless, both UBC and BBC have similar local control rates [3]. Thus, breast-conserving therapy, consisting of lumpectomy with adjuvant whole-breast definitive irradiation, has been demonstrated as an effective alternative to mastectomy in both UBC and BBC [3]. Whole bilateral breast irradiation was conventionally conducted with a three-dimensional conformal technique utilizing opposed tangential photon fields with minimal overlap at the sternal midline to mitigate additional morbidity [3,4]. 

Although mortality rates for BC have been decreasing in the US given advances in treatment [1], BBC has been demonstrated to have higher rates of distant disease and the worst disease-specific survival compared to UBC [2,5]. Breast cancer-specific mortality is a sequela of metastasis [5]. For metastatic breast cancer, there has been delineation between the oligometastatic state, with ≤ five depositions in ≤ three organs, and the polymetastatic state, given the reported differences in survival [4]. Oligometastatic breast cancer (OMBC) could benefit from metastasis-directed therapy (MDT), which is the multimodal combination of systemic therapy with a local therapy of radiation therapy or surgery for individual metastases [6]. This has been an active field of clinical investigation with Stereotactic Ablative Radiotherapy for the Comprehensive Treatment of Oligometastases (SABR-COMET) and NRG-BR002 randomized trials [6,7]. In short, these studies explore the promising role of radiotherapy in addition to systemic therapy for oligometastatic carcinomas in increasing overall survival (OS) [7].

We present a peculiar case of oligometastases of left breast IDC hormone receptor (HR) and epidermoid family growth receptor 2 (HER2) positive to the soft tissue of the sternum, an area originally spared during adjuvant radiation therapy while performing bilateral breast irradiation for original BCC a decade earlier. In this case, we also review the current MDT the patient is receiving in the form of hypofractionated radiotherapy and systemic therapy.

## Case presentation

A 53-year-old BReast CAncer gene 1 and 2 (BRCA1/2)-negative perimenopausal woman presented for multidisciplinary management of an oligometastatic, isolated recurrence of breast carcinoma in the soft tissue of the mid sternum. Her medical history was significant for estrogen receptor-positive (ER+), progesterone receptor negative (PR-), epidermoid family growth receptor 2 (HER2) unknown, left breast IDC, and right breast ductal carcinoma in situ (DCIS) 13 years ago. Additionally, the patient had an ER+/PR+/HER2 1+ left breast IDC recurrence 10 years ago.

Thirteen years ago, the patient was found to have clustered microcalcifications of the left breast on a screening mammogram that were correlated to a 1.5-cm hypodense lobulated mass at 12 o’clock on ultrasound (US). A US-guided biopsy of lesions demonstrated ER+/PR-DCIS arising from an intraductal papilloma with equivocal stromal invasion. In the workup, an incidental 1 cm nodule in the lower outer quadrant of the right breast was noted, and on a core needle biopsy, it proved to be a fibroadenomatoid tumor. A bilateral breast MRI scan did not demonstrate any further lesions. The patient underwent bilateral lumpectomies. A right breast excisional biopsy demonstrated a pTisNx DCIS of intermediate nuclear grade. A left breast lumpectomy demonstrated a 0.6 cm IDC with 0 out of six positive lymph nodes, thus staging as pT1bN0. It was determined that the tumor had an oncotype recurrence score (RS) of 22. The patient received adjuvant bilateral whole-breast radiation therapy. On the right breast, she received 6MV photon-based therapy of 5000 cGy in 25 fractions with no boost. On the left breast, she received 6MV photon-based therapy at 4860 cGy in 27 fractions with a cone down of 1,260 cGy in seven fractions with electrons. The patient was also recommended for adjuvant tamoxifen therapy but declined.

Ten years ago, in 2013, the patient had a recurrence of left IDC, for which the patient received a bilateral mastectomy with reconstruction. A 1.5cm x 0.7cm x 0.5cm pathological specimen with a positive posterior margin demonstrated ER95%/PR40%/HER1+, poorly differentiated oncotype type 33 left IDC with lymphovascular stromal invasion (LVSI). She received adjuvant chemotherapy, consisting of four cycles of doxorubicin and cyclophosphamide. The patient also completed five years of hormonal deprivation therapy with tamoxifen, which was successfully completed four years prior to the recurrence.

**Figure 1:**
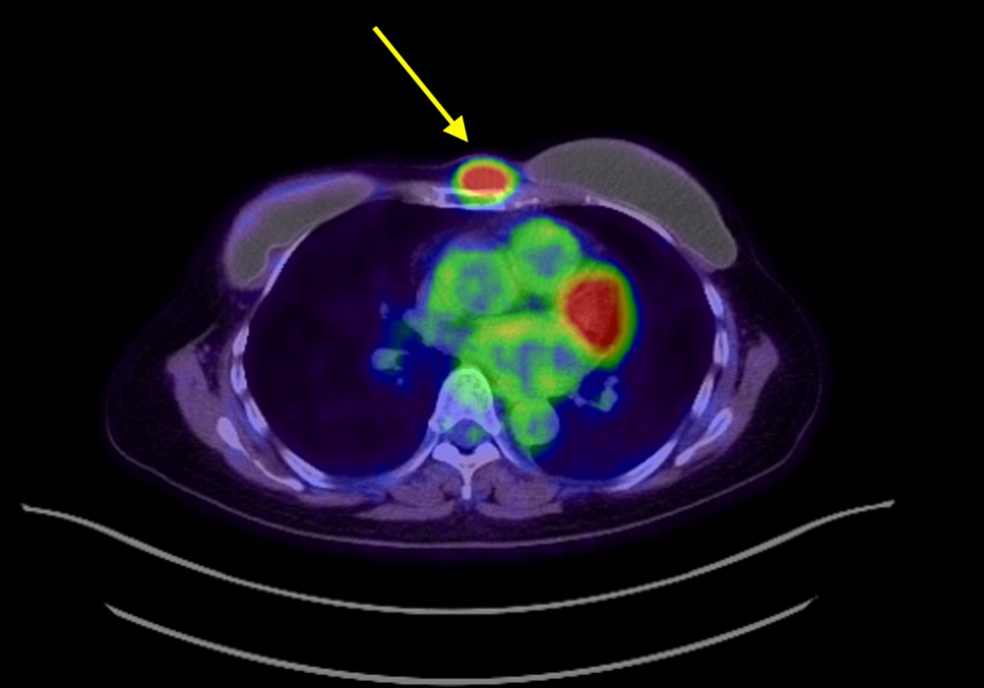
A positron emission tomography-computer tomography of left invasive ductal carcinoma recurrence in the soft tissue of the sternum The patient’s positron emission tomography-computed tomography (PET-CT) image demonstrates a hypermetabolic subcutaneous 2.37 cm x 1.16 cm PET avid nodule (yellow arrow) in the soft tissue of the anterior chest left to midline.

At the end of last year, the patient self-palpated a new onset of a growing painful mass over her sternal bone. A following positron emission tomography/computed tomography (PET/CT) scan demonstrated a hypermetabolic subcutaneous 2.37 cm x 1.16 cm nodule in the anterior chest left to midline (Figure 1).

On an excisional biopsy with clear margins, the sternal mass was determined to be ER>95%/PR 70%/Her2 2+ metastatic carcinoma, consistent with breast origin. The patient was diagnosed with either de novo breast cancer or OMBC and underwent evaluation for MDT. During the evaluation for radiation therapy, the patient’s previous dose distribution was reconstructed. To accomplish this, we fused the previous planning CT with the new planning CT, with a focus on the sternum. From this fused image, we transferred the previous dose distribution to the new planning CT. We were able to calculate the organs at risk from previous bilateral radiation. The lungs received an estimated mean dose of 972 cGy, and the volume of the lung receiving 2000 cGy (V20 Gy) was 17.3%. The heart received an estimated mean dose of 333 cGy, and the volume of the heart receiving 4500 cGy (V45 cGy) was 1.7%. The left anterior descending (LAD) artery received a maximum point dose (0.03) of 5363 cGy with a mean dose of 3543 cGy.

**Figure 2:**
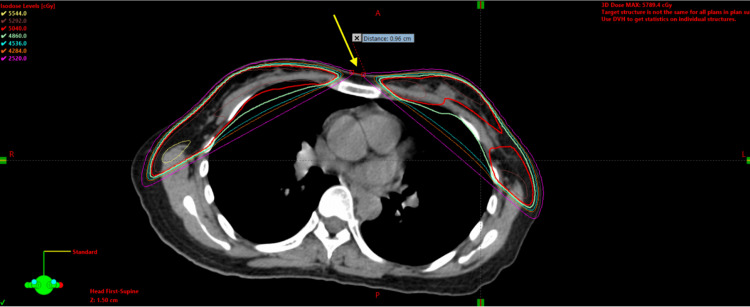
Reconstructed dose of radiation therapy tangents on the original computed tomography scan at the level of oligometastatic recurrence Composite dose of radiation therapy tangents from the original CT scan from 2010 at the level of the eventual sternal recurrence (yellow arrow). Of note, there is a gap measuring 0.96cm between tangential fields at the level of the oligometastatic recurrence.

During evaluation for radiation therapy, it was determined this neoplasm recurrence appeared within the “cold” spot of the previous bilateral whole breast irradiation. As such, we believe that this represents a distant metastatic recurrence; however, a capricious new hematogenous metastasis is also possible. The previous bilateral whole breast irradiation dose reconstruction at the level of the recurrence is shown below in Figure 2.

The patient was started on chemical ovarian suppression with Zoladex for 28 days in combination with letrozole and abemacicilib therapy. The patient will receive hypofractionated radiation therapy of 2500 cGy in five fractions using intensity-modulated radiation therapy to the soft tissue metastasis, given the sparing of the area originally. There was consideration of other organ-sparing techniques, such as electron beam plans. However, due to surface irregularities caused by the patient’s left breast impact, the target dose coverage was suboptimal. Special consent is also routinely executed in cases where tissue is being irradiated. In this case, the patient was told about potential long-term skin damage, such as atrophy, telangiectasis, fibrosis, and non-healing ulceration, although we feel that side effects would be unlikely as her skin lacked any evidence of prior morbidity.

**Figure 3:**
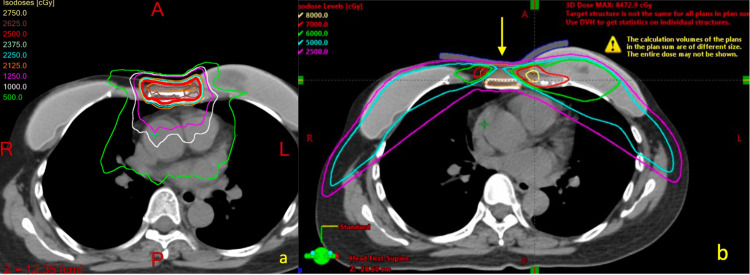
Current and composite dose of radiation therapy The oligometastatic soft tissue nodule will receive 25 Gy (a), which was spared during previous treatments, as demonstrated on the composite dose (b).

Likewise, care was taken to ensure that the regions of interest, i.e., the lung and heart doses, did not exceed tolerance. In our clinic, the left coronary artery is not digitalized for dose evaluation. The LAD artery will be receiving a maximum point dose (0.03 cc) of 763 cGy with a mean dose of 407 cGy. The current radiation plan and composite dosing are demonstrated below in Figure 3.

## Discussion

As both UBC and BBC have similar local control rates, breast-conserving therapy, consisting of lumpectomy with adjuvant whole-breast definitive irradiation, is offered as an effective alternative to mastectomy for patients with either UBC or BBC [3]. During this era, adjuvant radiotherapy for BCC following lumpectomy whole breast irradiation utilized conventional photon tangents [4,5]. Typically, doses included 45-60 Gy, with the primary tumor bed, commonly boosted to a total dose of 60-66 Gy [3], which is comparable to our patient’s treatment. When reconstructing the dosing on the original CT scan from 2010, our patient had a measured gap of 0.96 cm between tangential beams at the level where the eventual soft tissue sternal oligometastases occurred. A gap of tangential fields at the midline during bilateral whole breast irradiation for concurrent BBC is common practice in community settings and certain academic centers to prevent additional morbidity associated with sternal radiation [3]. We would agree that most radiation oncologists would not feel sternal metastasis to be likely after bilateral breast irradiation, but we do believe this particular case highlights the thin line between catastrophic treatment morbidity and underdosing our clinical tumor volume (CTV).

Interestingly, in the cases where there was an overlap of tangential fields at the sternum for patients, the majority had no evidence of disease at seven-year follow-up [3]. It is unclear if our patient’s recurrence after 13 years can be attributed to the cold spot during the original bilateral breast irradiation therapy. Nevertheless, our patient’s first treatment failure in the unilateral left breast was aligned with the natural course of BCC at the time, as was her therapy of mastectomy and salvage chemotherapy.

Now, 10 years later, OMBC is treated with curative intent using a combination of systemic therapy with the MDT approach of surgery or radiation at the location of the metastases [5,6]. Based on the findings of the SABR-COMET trial, there was a limited median progressive free survival (PFS) but significant five-year overall survival (OS) benefit for the addition of stereotactic radiation therapy to the standard of care systemic therapy for patients with oligometastatic carcinomas of less than five lesions [8]. However, recent phase II findings of the NRG-BR002 trial did not demonstrate such promise, with no OS or median PFS improvement detected for OMBC treated with MDT [9]. Although more studies are needed to fully appreciate the full benefits and limitations of MDT for OMBC, the most recent National Comprehensive Cancer Network (NCCN) guidelines for recurrent or stage IV disease do support the consideration of MDTs in the appropriate systemic therapy, particularly for locally recurrent disease [10]. Given that our patient has recurrent HR+ disease after breast-conserving therapy in an area previously not irradiated, local stereotactic radiotherapy to the sternal mass and a limited course of systemic therapy with an aromatase inhibitor with a CD4/6K is indicated [10]. The authors would like to note the fact that this patient only received five years of hormonal blockage as opposed to the current standard of 10 years. It is unknown whether additional blockades would have mitigated this recurrence.

Interestingly, from case series and retrospective studies, there appears to be a favorable prognosis for OMBC patients with isolated sternal metastasis treated with systemic therapy and MDT [6]. Likewise, systematic reviews have shown OMBC with certain features such as solitary metastasis, HR+, and disease-free interval > 24 months often have improved OS and PFS after MDT [6]. As our patient has an isolated HR+ soft tissue sternal metastasis greater than 24 months from the last recurrence, she is an ideal OMBC candidate for the MDT approach.

## Conclusions

We have detailed a peculiar case of left breast IDC with oligometastases to the soft tissue of the sternum, notably in an area not previously irradiated, over a decade from the original BCC diagnosis and treatment. This case also illustrates the application of MDT for OMBC, an evolving treatment paradigm. Future research is needed to assess the incidence and treatment for a local oligometastatic recurrence of bilateral breast cancer with no overlap of tangential photon fields during bilateral whole breast irradiation.
